# Selective breeding enhances coral heat tolerance even over small spatial scales

**DOI:** 10.1098/rspb.2025.1817

**Published:** 2025-10-01

**Authors:** Alexandra Kler Lago, Kate Kiefer, Marie E. Strader, Teresa Baptista Nobre, Stephanie F. Hendricks, Claudio Richter, Christian Wild, Kate M. Quigley

**Affiliations:** ^1^ University of Bremen, Bremen 28359, Germany; ^2^ Minderoo Foundation, Perth 6009, Australia; ^3^ Department of Biology, Texas A&M University, College Station, TX 7853-3258, USA; ^4^ University of Western Australia, Perth 6009, Australia; ^5^ Alfred-Wegener Institute Helmholtz Centre for Polar and Marine Research, Bremerhaven 27570, Germany; ^6^ James Cook University, Queensland 4811, Australia

**Keywords:** coral reefs, heat stress, restoration, marine heatwaves, climate change

## Abstract

Coral reefs globally are experiencing escalating mass bleaching and mortality. Reefs along the Western Indian Ocean have been relatively unimpacted. We established heat tolerance baselines and selective breeding efforts for two widespread reef-building *Acropora* species within the Ningaloo World Heritage Area. To accomplish these goals, we included corals from two thermally distinct southern and northern reefs (mean monthly maximum 27.9°C and 26.6°C, respectively), which reflect both present-day and stress histories. Fitness responses were measured in control and heat stress temperatures (adults = 31.0°C; larvae = 35.5°C), including survival, tissue necrosis, bleaching and photosynthesis. Larvae with one parent from the warmer population exhibited >2.2-fold higher survival under heat stress, while those with both parents from the warmer population survived 1.6-fold better (compared with control larvae with two parents from the cooler population). Photosynthesis was maintained in both species and both populations, suggesting heat responses were host-driven. Adults from both populations of one species (*Acropora tenuis*) exhibited similar responses to heat, while the other (*Acropora millepora*) was more variable. These findings are the first to demonstrate that selective breeding can provide heat tolerance enhancement for corals in the Indian Ocean and will be critical to preparing for future marine heatwaves.

## 1. Background

Since the pre-industrial era (approximately 1850–1900), coral reefs have experienced four global mass bleaching events [1]. This has caused the global decline of approximately 50% in coral cover, mainly attributed to an, on average, +1°C of ocean warming and intensified marine heatwaves (MHWs) [2]. Within the next three decades, climate predictions strongly indicate an increase in ocean surface temperatures by +1.5 to +2.0°C, accompanied by warmer (+1.9 to +2.5°C), prolonged and more frequent (4.1× to 5.6×) MHWs per decade [3,4]. This warming will likely result in annual mass bleaching across most reefs, with a projected 70 to 99% loss of coral cover and potential ecological collapse beyond +2.5°C warming [3,4]. Such extensive mortality indicates that the rate of temperature increase may be outpacing the natural rate of thermal adaptation in corals, which will impact coral populations’ recovery and replenishment [5,6]. Given coral reefs’ ecological and economic importance [7], active interventions beyond conventional reef management and restoration approaches are urgently needed [8]. These actions should bolster resilience to maintain critical ecosystem functions and services until greenhouse gas emissions and warming are brought under control.

Emerging active intervention tools like assisted evolution have been proposed to accelerate adaptation by increasing heat tolerance in corals and their symbionts faster than natural rates [9,10]. These tools include selective breeding of the coral host [11], which consists of selecting and reproductively crossing coral parental stocks with heritable fitness-related traits associated with higher heat tolerance to enhance these same traits in the offspring. While there are multiple approaches to selecting parental stocks, which vary with scalability, costs and technical dependency, most studies have used local summer maxima temperatures and/or bleaching responses as proxies for heat tolerance in parental stocks for breeding [12]. Another study also found specific historical temperature, daily temperature variation and thermal stress metrics for predicting selection of thermally tolerant parental stocks [13]. The end goal is to increase desired traits (e.g. increased heat tolerance) while maintaining the genetic diversity of local populations. When combined with movement, transferring selected offspring to at-risk reef locations—a process known as assisted gene flow—can improve the resilience of coral populations within their known ranges [14].

Selective breeding studies, which have been performed on the Great Barrier Reef (GBR), Persian Gulf, Hawai’i and Palau, have achieved promising results in selected offspring (i.e. larvae and juveniles) with an increased survivorship to warming [15–17]. This adaptive response may be driven by a substantial genetic component to heat tolerance. Narrow-sense heritability (*h*^2^) estimates for heat tolerance have been consistently high, exceeding 0.2 [16,18–20]. Importantly, the heritability of many coral traits relevant to temperature tolerance is considerably heterogeneous across stages/ages, growth forms and environments [21], as seen, for example, in survival under heat stress between *Acropora* corals in Palau (*h*^2^ = 0.2–0.3) and the GBR (*h*^2^ = 0.9) [16,20]. This variation underscores the importance of generating baseline information on heat tolerance and heritability across diverse coral populations for the development of targeted selective breeding strategies.

In the last three decades, the Ningaloo World Heritage Area in Western Australia (WA), one of the world’s most remote, diverse and extensive fringing reef systems spanning approximately 290 km, has experienced five recorded severe heat stress events (degree heating weeks (DHWs) >8°C-weeks) since 1998 [22,23]. This includes the severe 2011 MHW, which resulted in sea surface temperatures (SSTs) up to 5°C warmer than average for more than 10 weeks [24,25]. Ningaloo harbours half of the coral species in the Indian Ocean [26], making it an outstanding ark of global coral biodiversity and a target for conservation priority under the risk of annual bleaching by 2050 [27,28]. During these past heat stress events, coral bleaching and mortality responses have been spatially variable, with some reports showing an up to 92% and 32% decline in the northeastern and southern areas of Ningaloo, respectively, while coral cover remained stable in the northern region [22,29–31]. These spatial differences in bleaching severity may reflect significant variation in local thermal regimes and historical exposure to heat stress between the northern and southern regions in the Ningaloo Coast. Despite these patterns, baselines in coral heat tolerance have not yet been determined, and it is unclear how vulnerable these populations are to climate change. Furthermore, no selective breeding studies have been conducted in this globally important region.

**Figure 1.**
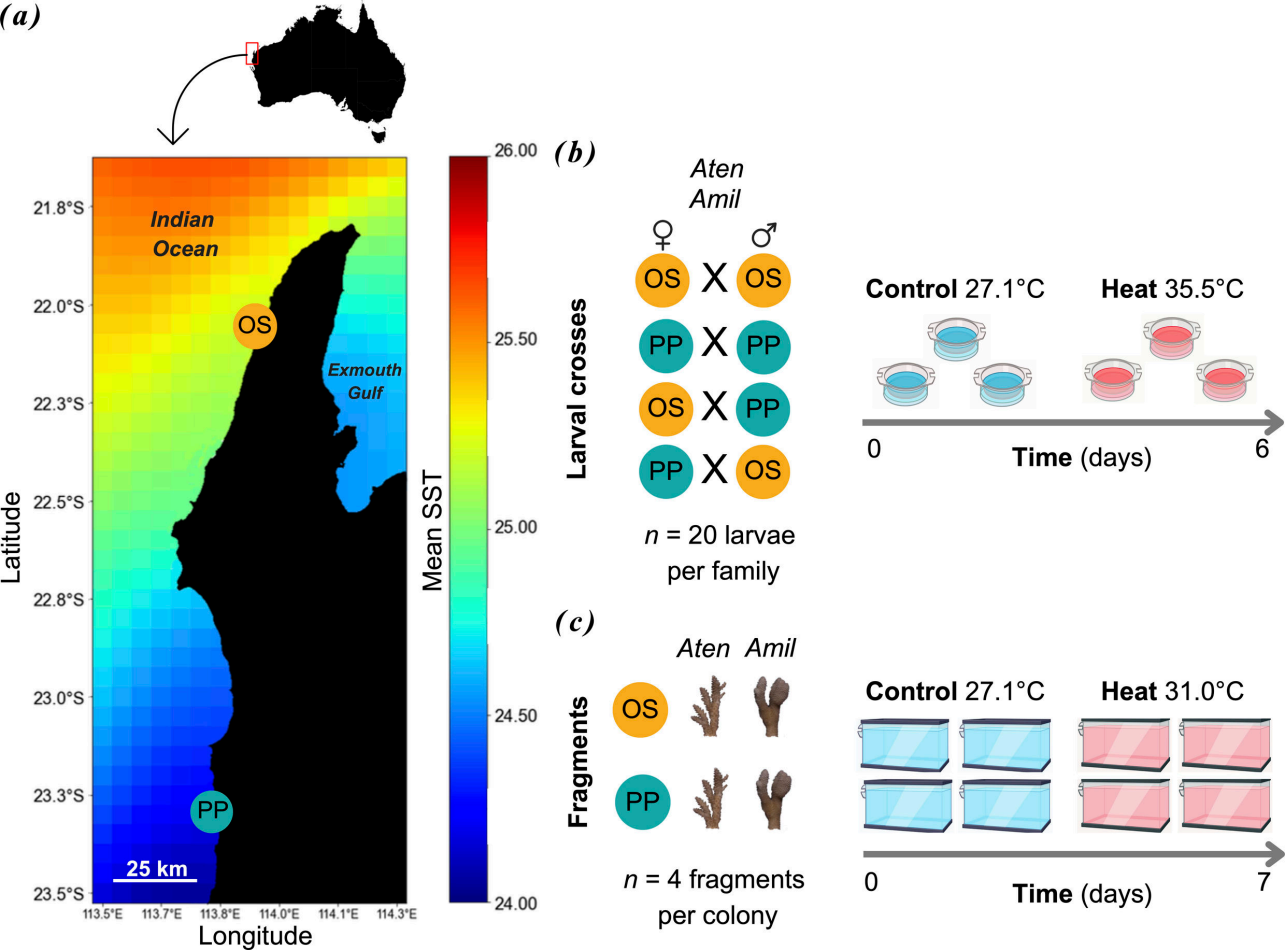
Experimental design for adult and larval heat stress. (*a*) Mean annual SSTs along the Ningaloo Coast (1985−2022; NOAA CoralTemp). Collection sites of *Acropora tenuis* (Aten) and *Acropora millepora* (Amil) at Oyster Stacks (OS, yellow) and Pelican Point (PP, blue). (*b*) Larval heat stress: 20 larvae per family from intrapopulation (OS × OS, PP × PP) and interpopulation (OS × PP, PP × OS) crosses were tested at 27.1°C and 35.5°C. (*c*) Adult heat stress: 3−4 fragments per genotype from both populations were tested at 27.1°C and 31.0°C with daily assessments of survival, necrosis, bleaching and photochemical efficiency.

To address these knowledge gaps, we conducted assessments of selective breeding feasibility and heat tolerance for two common and widespread *Acropora* species sourced from the warmer, northern (Oyster Stacks, OS) and cooler, southern (Pelican Point, PP) regions of the Ningaloo Coast. These two locations on the reef were selected for their distinct thermal profiles (figure 1). We tested and quantified four fitness-related responses—specifically, survival, tissue necrosis, bleaching and photochemical efficiency (Δ*F*/*Fm*′)—in adult (parental) corals and survival in their selectively bred aposymbiotic larval offspring under experimental heat stress. We assessed responses of larval offspring produced from warmer northern (OS) and cooler southern (PP) parent corals, as well as reciprocal crosses with mixed parents. Based on previous studies using selective breeding in corals, we expected that offspring with at least one parent from a warmer reef would exhibit increased survival under heat stress. Overall, our study demonstrated that selective breeding significantly improved the heat tolerance of an early life-history stage in the two *Acropora* coral species by up to 2.2-fold, even though adult responses to heat stress were variable.

## 2. Results

### (a) Distinct thermal profiles between reefs in historical and heat stress periods

Temperature metrics related to SST (annual mean and variation) and thermal anomalies (frequency of anomalies and cumulative heat stress) were assessed for the warmer, northern site (OS) and cooler, southern site (PP) over two time periods: a historical baseline (1985−2010) and a post-MHW period (2010−2022), which includes the 2011−2013 heat stress events.

**Figure 2.**
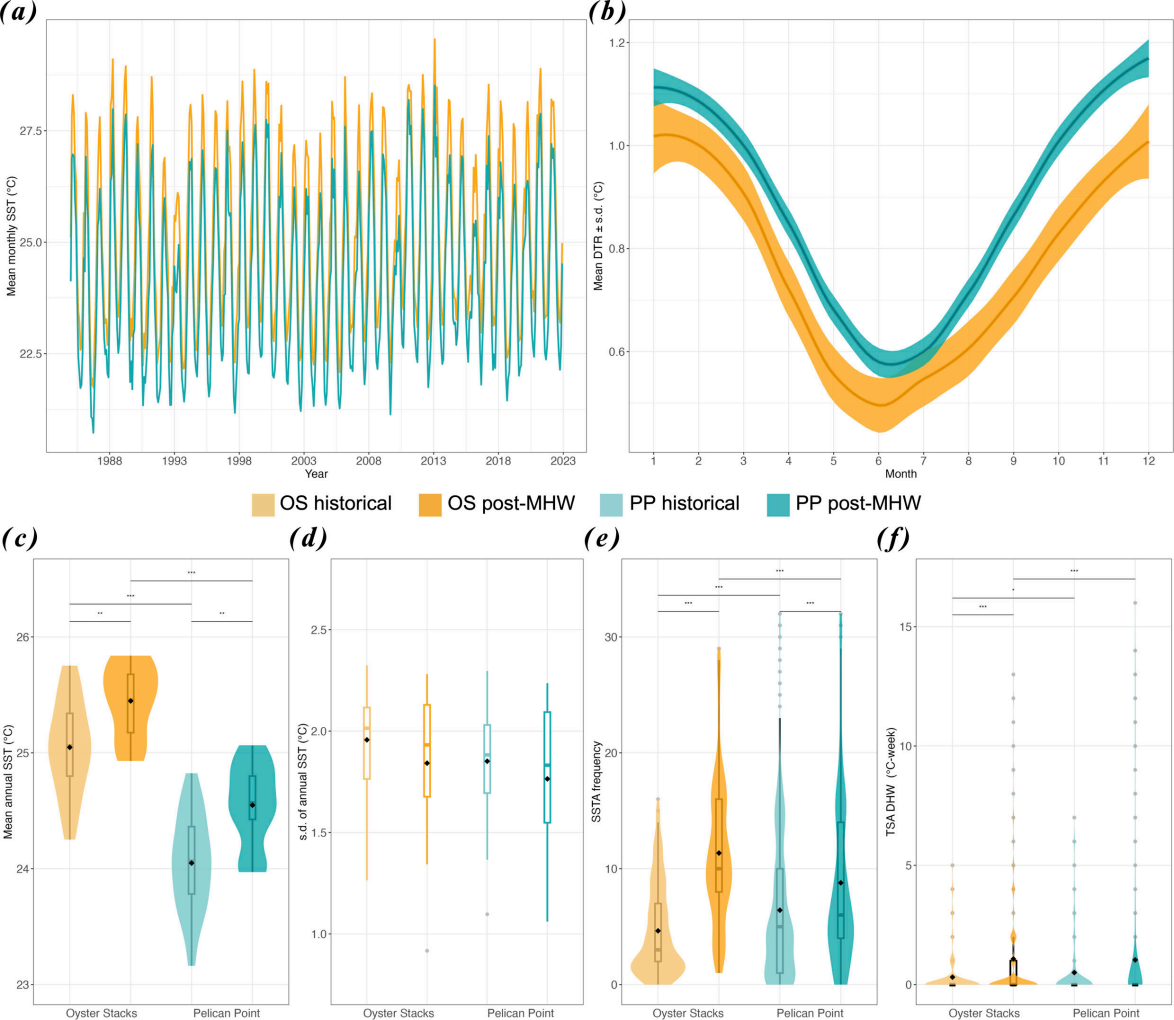
Temperature metrics of collection sites on the Ningaloo Coast. (*a*) Mean monthly SST time series (1985−2023) for Oyster Stacks (OS, yellow) and Pelican Point (PP, blue). (*b*) Diurnal temperature range ± standard deviation (DTR ± s.d.) over a 12 month cycle (2016−2022). (*c*) Mean annual SST (SST_mean) for historical (1985−2010, pale) and post-MHW (2010−2022, dark) periods. Panels (d–f) show annual SST s.d. (SST_stdev), SST anomaly frequency (SSTA_Freq) and cumulative heat stress in DHWs (TSA_DHW). Boxplots display medians (centre lines), quartiles (box limits), 1.5× interquartile range (whiskers) and outliers (points); diamonds indicate means. Horizontal lines with asterisks represent significant differences (*p < 0.05, **p < 0.01, ***p < 0.001) between locations and periods (Wilcoxon’s test); *p*-values are detailed in the electronic supplementary material, table S5.

Overall, OS was consistently approximately 1°C warmer than PP across both time periods (figure 2*a*,*c*). The mean annual temperature at OS was 25.0 ± 0.4°C (mean ± s.d.) and 24.0 ± 0.4°C at PP during the historical period, increasing to 25.4 ± 0.3 °C and 24.5 ± 0.4 °C, respectively, in the post-MHW period (Wilcoxon, both *p* < 0.001). Maximum monthly mean (MMM) temperatures also increased post-MHW, reaching 28.1 ± 0.7°C at OS and 26.9 ± 0.8°C at PP. OS experienced significantly higher cumulative thermal stress post-MHW (figure 2*e*; Wilcoxon, *p* < 0.001). While annual SST variability was similar across sites and periods (figure 2*d*; Wilcoxon, *p* > 0.5), PP exhibited a significantly higher diurnal temperature range (figure 2*b*; *p* < 0.001). Both sites experienced a marked increase in the frequency of SST anomalies over time (OS: +2.5×; PP: +1.7×; both Wilcoxon, *p* < 0.001). Full results are provided in electronic supplementary material, text S1 and tables S1–S3.

### (b) Differences in selected larval survival responses to heat stress between reproductive crosses

Larval survival in both *Acropora* species was measured across 44 unique families produced from the intrapopulation crosses from the cooler northern (PP × PP) and warmer southern (OS × OS) reefs, respectively. Interpopulation crosses (OS × PP and PP × OS) were also produced, with the maternal colony listed first. The cross OS × OS for *A. millepora* could not be performed and is therefore not included in this section. Survival was assessed in three replicates of 20 larvae per family per treatment throughout the experimental period.

**Figure 3.**
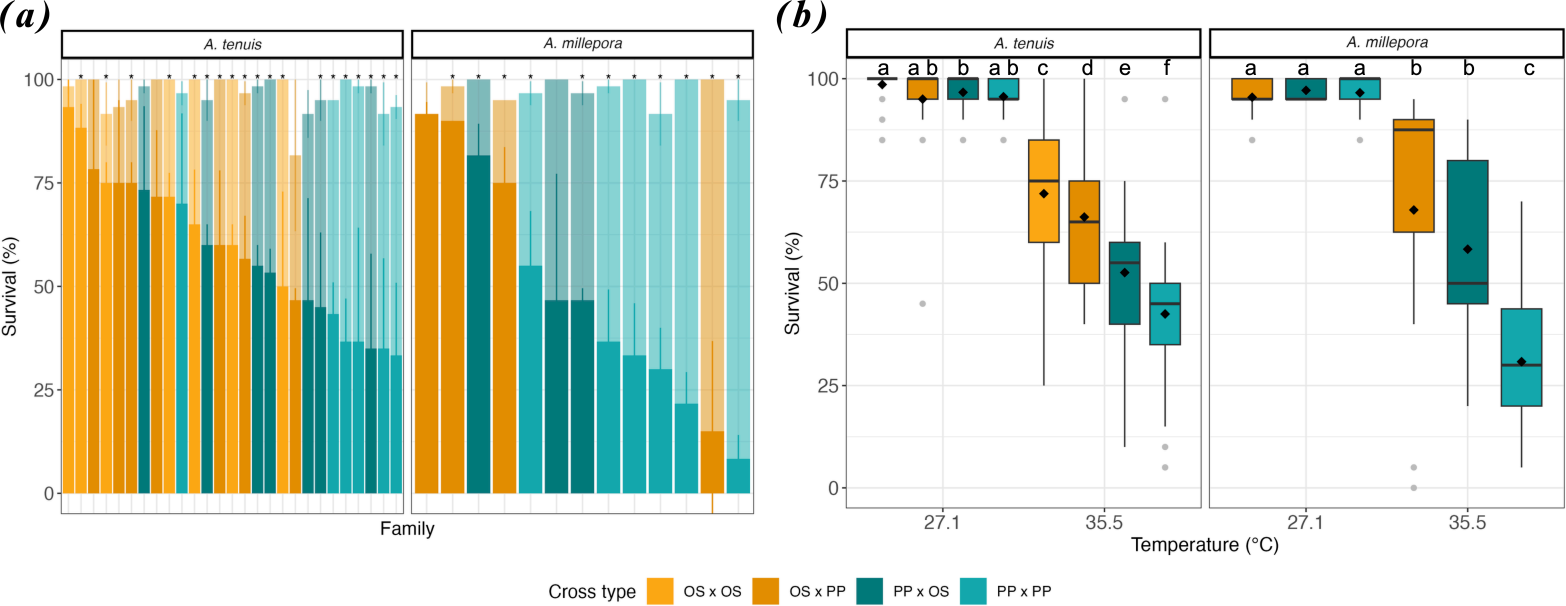
Survival responses in selected larvae under heat stress. (*a*) Variation in per cent survival (M ± s.e.) among *Acropora tenuis* (*n* = 3320) and *A. millepora* (*n* = 1560) larval families from the intrapopulation crosses within the warmer northern (OS × OS) and cooler southern (PP × PP) reefs, as well as interpopulation crosses (OS × PP and PP × OS) after 143 and 93 h of exposure to 35.5°C (foreground) versus 27.1°C (background). Note the OS × OS cross is absent for *A. millepora* due to the limited availability of unique gametes. (*b*) Median survival in larval crosses exposed to control (27.1°C) and heat (35.5°C) treatments. Boxplots show medians (centre lines), quartiles (box limits), 1.5× interquartile range (whiskers) and outliers (points); diamond shapes indicate the mean survival. Asterisks indicate significant differences between treatments using one-tailed *t*-tests with adjusted *p*-values (electronic supplementary material, table S7). Letters denote significant differences across all treatment combinations within each species based on KM log-rank tests with Bonferroni-corrected *p*-values (electronic supplementary material, table S8).

*Acropora tenuis* showed a significant difference in larval survival for most families produced (electronic supplementary material, table S7 for *t-*test *p*-values) and for all reproductive crosses between temperature treatments after 6 days of experimental heat stress (figure 3*a*,*b* and electronic supplementary material, figure S3*a*–S3*d* and table S8; log-rank Kaplan–Meier (KM), *p*-values < 0.001). In the control treatment, there was no difference in *A. tenuis* larval survival between crosses, except for one (electronic supplementary material, table S8; log-rank KM, *p* = 0.160 for OS × OS versus OS × PP). However, in heat conditions, larval survival differed significantly between all crosses (electronic supplementary material, table S8; log-rank KM, *p* < 0.001 for all pairwise comparisons). In heat conditions, *A. tenuis* larvae from intrapopulation crosses recorded a mean endpoint survival of 71.9 ± 17.3% (OS × OS) and 44.7 ± 19.0% (PP × PP), while those from interpopulation crosses were 66.2 ± 4.1% (OS × PP) and 52.6 ± 4.0% (PP × OS), which were 1.4× to 2.2× lower compared with controls (OS × OS: 98.6 ± 3.9%, PP × PP: 96.1 ± 4.7%, OS × PP: 98.3 ± 1.1%, PP × OS: 96.7 ± 0.9%; figure 3*b*). Relative to the intrapopulation PP × PP larvae, which recorded the lowest mean endpoint survival, intrapopulation OS × OS larvae had the highest survival gain by 1.6× (+27.2%), followed by interpopulation crosses OS × PP with 1.5× (+21.5%) and PP × OS with 1.2× (+7.9%) under heat conditions (figure 3*b*). Variation in survival within larval families (compared with between them) was also substantial at the final time point. For example, although most PP × PP and PP × OS families exhibited low mean survival (<50% and <60% at heat, respectively), one family within each group showed high survival (70% in PP × PP and 73.3% in PP × OS, respectively; figure 3*a*).

For *A. millepora,* there was also a significant effect of temperature in larval survival for most families (electronic supplementary material, table S7 for *t-*test *p*-values) and for all reproductive crosses after 4 days of heat exposure (figure 3*a*,*b* and electronic supplementary material, figure S3e–S3g and table S8; log-rank KM, *p-*values < 0.001). In the control, there was no difference in *A. millepora* larval survival between all crosses (see electronic supplementary material, table S8 for *p*-values). In comparison, at heat, there were significant differences in *A. millepora* larval survival between intrapopulation and interpopulation crosses (electronic supplementary material, table S8; log-rank KM, *p* < 0.001 for all pairwise comparisons) but not between interpopulation crosses (electronic supplementary material, table S8, log-rank KM, *p* = 1.000 for OS × PP versus PP × OS). In heat conditions, *A. millepora* intrapopulation cross PP × PP recorded a mean endpoint survival of 30.8 ± 4.1%, while interpopulation crosses OS × PP and PP × OS had 67.9 ± 9.9% and 58.3 ± 7.9%, respectively, which were 1.9× to 3.2× lower compared with the control (PP × PP: 97.8 ± 1.4%, OS × PP: 96.3 ± 1.5%, PP × OS: 100.0 ± 2.2%; figure 3*b*). Relative to the intrapopulation PP × PP larvae with the lowest mean survival, interpopulation crosses had a survival gain of 2.2× (+37.1%) and 1.9× (+27.5%; figure 3*b*). Despite the overall patterns, substantial variation among families was observed at the final time point. Although most OS × PP families had high mean survival under heat stress (>75%), one family showed unexpectedly low survival (15%), comparable to the lowest-performing PP × PP cross. Conversely, while most PP × OS had a mean survival <50%, one family exhibited high survival (81.2%), exceeding the OS × PP mean (figure 3*a*).

Estimated DHW experienced during the larval heat stress was 6.3 and 7.3 for *A. tenuis* and 4.1 and 4.8 for *A. millepora*, for OS and PP, respectively. Using estimates of projected end-of-century MMMs for OS and PP resulted in estimated DHW to be 5.1 and 6.1 for *A. tenuis* and 3.3 and 4.0 for *A. millepora*, at OS and PP, respectively (electronic supplementary material, table S11).

### (c) Differences in adult physiological responses to heat stress between reefs

Physiological responses to heat stress were measured in both *Acropora* species from warmer, northern and cooler, southern populations (OS and PP, respectively), including the parental colonies used in the reproductive crosses. Responses were assessed using three to four replicates of each colony per population in each treatment throughout the experimental period.

### (d) Survival

**Figure 4.**
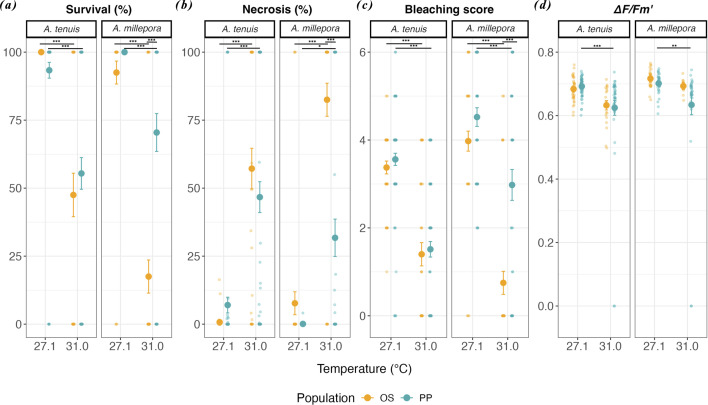
Physiological responses of adult coral fragments under heat stress. Endpoints of (*a*) per cent survival, (*b*) per cent necrosis, (*c*) bleaching score and (*d*) effective quantum yield (∆*F*/*Fm*′) in *Acropora tenuis *(*n* = 229) and *Acropora millepora *(*n* = 168) adult fragments from the warmer northern reef (Oyster Stacks, OS in yellow) and cooler southern reef (Pelican Point, PP in blue) reefs after 7 days of exposure to control (27.1°C) and heat (31.0°C) treatments. Fragments include both spawning parental colonies and colonies not used in selective breeding. Dot shapes represent means ± standard errors. Horizontal lines with asterisks denote significant differences (*p < 0.05, **p < 0.01, ***p < 0.001) determined by KM log-rank tests for survival and GLMMs Tukey *post hoc* tests for other responses. Adjusted *p*-values (Bonferroni correction) are detailed in the text.

After 7 days of heat exposure for adult *A. tenuis* fragments, there was a significant effect of temperature on the survival probabilities across populations (electronic supplementary material, figure S2*a,b* and table S8; log-rank KM, *p* < 0.001 for OS and PP). However, there was no population origin effect on the survival of *A. tenuis* fragments in either temperature treatments (electronic supplementary material, table S8; log-rank KM, *p =* 0.086*, p* = 0.613 for control and heat, respectively). In the heat treatment, OS fragments survived on average 2.1× less (47.5 ± 50.6%) and PP fragments 1.7× less (55.4 ± 50.0%) compared with fragments in the control (OS: 100.0 ± 0%, PP: 93.3 ± 25.1%; figure 4*a*).

For *A. millepora* fragments, temperature also had a significant effect on the survival probability for each population at the end of the heat stress treatment (electronic supplementary material, figure S2*c*,*d* and table S8; log-rank KM, *p* < 0.001 for OS and PP). Moreover, population origin had a significant effect on the survival probability of *A. millepora* fragments in the heat treatment (electronic supplementary material, table S8; log-rank KM, *p* < 0.001). At the end of the heat exposure, OS fragments survived on average 5.7× less (17.5 ± 38.4%) and PP fragments 1.3× less (70.5 ± 46.2%) than fragments in the control (OS: 100.0 ± 0%, PP: 92.5 ± 26.7%; figure 3*a*). Between populations, PP fragments had a mean endpoint survival at heat 4.0× higher than that of OS fragments (figure 4*a*).

### (e) Tissue necrosis

For *A. tenuis* fragments, there was a significant difference in tissue necrosis owing to temperature across populations at the endpoint of heat exposure (figure 4*b* and electronic supplementary material, tables S9 and S10; Tukey *post hoc* beta generalized linear mixed model (GLMM), *p* < 0.001 for OS and PP). However, population origin did not have a significant effect on necrosis in both temperature treatments (figure 4*b* and electronic supplementary material, tables S9 and S10; Tukey *post hoc* beta GLMM, *p* = 1.000 for heat and control). Relative to average tissue loss in the control (OS: 0.7 ± 3.1%, PP: 7.0 ± 25.1%), OS fragments of *A. tenuis* lost 78.4× more tissue (57.2 ± 47.3%), whereas PP fragments experienced 6.7× more tissue loss (46.7 ± 48.8%) under heat stress (figure 4*b*).

Similarly, for *A. millepora*, temperature had a significant effect on necrosis of fragments across populations at the endpoint of heat exposure (figure 4*b* and electronic supplementary material, tables S9 and S10; Tukey *post hoc* beta GLMM, *p* < 0.001 for OS and *p* = 0.035 for PP). Tissue loss of fragments also differed significantly between the two populations in the heat treatment (figure 4*b* and electronic supplementary material, tables S9 and S10; Tukey *post hoc* beta GLMM, *p* < 0.001). At the end of the heat treatment, OS fragments of *A. millepora* recorded on average 10.7× more tissue loss (82.5 ± 38.5%), whereas PP fragments lost 353.3× more tissue (31.8 ± 45.6%) than those in the control (7.7 ± 26.7% for OS, 0.1 ± 0.6% for PP; figure 4*b*). Between populations, PP fragments recorded 2.6× less tissue loss than those from OS at heat (figure 4*b*).

For the fragments that died during the experiment, these fragments exhibited rapid tissue loss from 0 to 100% between 24 and 48 h of experimental heat stress; however, averages and differences in time to complete tissue loss were not incorporated above (electronic supplementary material, figure S4).

### (f) Bleaching

After *A. tenuis* fragments were exposed to heat stress, bleaching scores were significantly different due to temperature (figure 4*c* and electronic supplementary material, tables S9 and S10; Tukey *post hoc* negative binomial (nb) GLMM, *p* < 0.001) but not due to population origin (figure 4*c* and electronic supplementary material, tables S9 and S10; Tukey *post hoc* nb GLMM, *p* = 1.000). At heat, OS fragments bleached, on average, 2.4× more (1.4 ± 1.7) and PP fragments 2.4× more (1.5 ± 1.5) than those in the control (OS: 3.4 ± 0.9, PP: 3.6 ± 1.2; figure 4*c*).

For *A. millepora*, there was a significant effect of both temperatures (figure 4*c* and electronic supplementary material, tables S9 and S10; Tukey *post hoc* nb GLMM, *p* < 0.001 for OS and *p* = 0.001 for PP) and population origin (figure 4*c* and electronic supplementary material, tables S9 and S10; Tukey *post hoc* nb GLMM, *p* < 0.001) on the mean bleaching scores of fragments at the endpoint of heat stress exposure. Relative to bleaching in the control (OS: 4.0 ± 1.4, PP: 4.5 ± 1.4), OS fragments bleached, on average, 5.3× more (0.8 ± 1.7), whereas PP fragments 1.5× more (3.0 ± 2.3) in the heat treatment (figure 4*c*). Between populations, PP fragments recorded 3.9× higher bleaching scores compared with OS fragments at heat (figure 4*c*).

### (g) Photochemical efficiency

For *A. tenuis*, the photochemical efficiency response, as measured by the effective quantum yield (∆*F*/*Fm*′), only decreased significantly for PP fragments in the heat treatment compared with the control at the endpoint (figure 4*d* and electronic supplementary material, tables S9 and S10; Tukey *post hoc* GLMM, *p* < 0.001). Mean yields at heat were 1.1× lower relative to control conditions (figure 4*d*). Reef origin was not a significant factor in explaining differences in ∆*F*/*Fm*′ across temperature treatments (figure 4*d* and electronic supplementary material, tables S9 and S10; Tukey *post hoc* GLMM, *p* = 1.000 in control and heat). At the experiment endpoint, fragments across populations recorded similar mean yields under heat (OS: 0.6 ± 0.1, PP: 0.6 ± 0.2) and control conditions (OS: 0.7 ± 0.0, PP: 0.7 ± 0.0; figure 4*d*).

Similar to *A. tenuis*, only PP fragments of *A. millepora* had a significant difference in their ∆*F*/*Fm*′ due to temperature at the endpoint of heat stress exposure (figure 4*d* and electronic supplementary material, tables S9 and S10; Tukey *post hoc* GLMM penalized quasi-likelihood (PQL), *p* = 0.004). Also, the population origin effect on the ∆*F*/*Fm*′ of fragments was not significant (figure 4*d* and electronic supplementary material, tables S9 and S10; Tukey *post hoc* GLMM PQL, *p* = 1.000). Endpoint yields were similar across populations in both temperature treatments (OS: 0.7 ± 0.0 at heat, 0.7 ± 0.0 at control, PP: 0.7 ± 0.0 at heat, 0.6 ± 0.2 at control; figure 4*d*).

In addition to these population-level analyses, which examined adult coral responses across all colonies collected, we also undertook analyses that examined responses in only corals that were parents to offspring. We did this because spawning parents only represented a subset of the adult colonies that were measured. Overall, we found that restricting the adult dataset to only parental colonies did not substantially alter the interpretation of these data (electronic supplementary material, figure S5). *A. tenuis* parental colonies from PP demonstrated overall improved heat stress responses, but with considerable variability between individuals, as expected. Specifically, these colonies survived better and exhibited less necrosis and bleaching compared with OS. Photo-physiology remained unchanged. Compared with the larger dataset, PP parental colonies having an overall improved response to heat is more evident. Moreover, like in the larger dataset, adult responses still do not align with the larvae responses. Trends for *A. millepora* parental colonies were even more consistent with the full dataset. Taken together, there does not appear to be a clear divergence from the patterns we observed between the full population-level dataset compared with the spawning-only parental colonies.

## 3. Discussion

This study presented the first evidence for enhanced heat tolerance in *Acropora* offspring using only one generation of selective breeding at the Ningaloo World Heritage Area. These results confirm the feasibility of selective breeding along even a small but significant thermal gradient. Interestingly, coral adults exhibited a more complex response to heat stress, marked by less variation between warmer and cooler populations, and driven primarily by host-related processes and with less response from their symbionts, although we did not undertake a direct comparison of any metrics between host and symbiont compartments.

### (a) Evidence of heat tolerance boost in *Acropora* larvae

Adaptation can occur in response to selection imposed by local environmental conditions, particularly thermal regimes, across different spatial scales [32]. The resulting standing genetic variation in coral populations is critical for selectively breeding corals for fitness-related traits [33]. In this study, we reported 1.6× greater heat tolerance among *A. tenuis* intrapopulation larvae from the warmer northern reef compared with those from the cooler southern reef (figure 2*a*,*b*). These results demonstrate a significant difference in larval heat tolerance across <100 km and a temperature differential of approximately 1°C in mean annual and MMM temperatures—less than the average 2°C reported in previous selective breeding experiments [11,16,34,35]. In this context, selective breeding using northern parental stock, sourced from a location of significantly higher mean annual temperatures and more frequent and intense recent thermal stress since the 2011 MHW compared with southern corals, will likely be effective in enhancing heat tolerance in this early life-history stage in the short term.

Selective breeding has been shown to significantly increase heat tolerance in critical early life-history stages of corals by mixing gametes from corals sourced from different thermal environments [15]. Consistent with these previous findings and building from the reported difference in heat tolerance between northern and southern larvae, we found that crossing gametes of one parent from a warmer and more thermally impacted reef with a cooler, less impacted reef yielded larvae with up to 1.5-fold (*A. tenuis*) or 2.2-fold (*A. millepora*) higher heat tolerance compared with the control larvae with both parents sourced from the cooler, less impacted reef (figure 3*a*,*b*). When expressed in terms of cumulative heat stress exposure, the observed survival at 35.5°C indicates that larvae can withstand an additional 6.3−7.3 DHW (*A. tenuis*) and 4.1−4.8 DHW (*A. millepora*). Using MMMs beyond the projected end-of-century summer maxima under a moderate emissions scenario (representative concentration pathway (RCP) 4.5, +1.4°C, [36]; electronic supplementary material, table S11), expected DHWs would be between 5.1−6.1 DHW (*A. tenuis*) and 3.3−4.0 DHW (*A. millepora*). These results align with previous studies showing that heat tolerance is a heritable trait [16,17,19,20,35,37] and that one cycle of selective breeding can yield offspring survival gains of 20–70% across species such as *Acropora, Montipora* and *Platygyra*, primarily in the GBR and Persian Gulf [15]. Importantly, our findings, together with recent studies demonstrating a heat tolerance enhancement within individual reefs in Hawai’i and Palau [16,38,39], confirm that measurable improvements in the heat tolerance of this early life stage can be achieved even across very fine spatial and thermal gradients. Combined with breeding, these results support that assisted gene flow could be used as a conservation strategy to enhance coral resilience while maintaining the local genetic diversity along the Ningaloo Reef.

### (b) Species-specific parental effects on larval heat tolerance

Parental genotypes play a crucial role in shaping offspring fitness and stress responses, including heat tolerance, where both the maternal and paternal identities influence offspring fitness [40–42]. In our study, we found significant species-specific parental effects on the heat tolerance of interpopulation coral offspring. For *A. tenuis*, maternal colonies from the warmer reef conferred greater heat tolerance to offspring than paternal colonies from the same environment (figure 3*b*), aligning with a previous study on maternal influence on survival and symbiont acquisition in *Acropora* [43]. In contrast, *A. millepora* exhibited no difference in heat tolerance between offspring with maternal or paternal colonies from the warmer reef (figure 3*b*). Other studies have shown regional and species variations in parental effects on heat tolerance, including strong paternal effects in *Platygyra* from the Persian Gulf [19,37] and pronounced maternal effects in *Montipora* from Hawai‘i [39,44]. Taken together, these findings suggest that parental contributions to heat tolerance are highly species and population specific and are likely influenced by both environmental and genetic factors. Importantly, they highlight that conservation strategies will likely need to be tailored to specific species. This emphasizes the need for selective breeding programmes that consider species-specific variability when optimizing strategies to enhance coral tolerance to climate change.

### (c) Distinct heat tolerance between adult corals and selected larvae

Following heat stress events between 2011 and 2013, coral populations in northern Ningaloo were more impacted and showed greater recovery compared with southern populations [22,31,45]. This suggests that northern populations have already undergone some selection for increased tolerance due to disturbance and could explain the higher tolerance transfer from northern parents to offspring, even if northern and southern adults showed similar heat tolerance. This difference between adult and offspring tolerance may be due to their symbiotic states (aposymbiotic larvae and symbiotic adults) and is not necessarily surprising, given that it aligns with findings where adult *Acropora* GBR populations showed reduced variation in tolerance compared with their selected offspring [35]. Other studies also found variable stress responses across life-history stages—with less tolerant parents yielding more tolerant offspring [46–48]. Other factors, like symbiosis, transgenerational plasticity and parental provisioning (e.g. lipids) play a significant role in determining heat tolerance aside from the host genetics [34,49–51]. Differences between larvae and adults here suggest that symbionts and non-genetic effects may play an important role in determining heat tolerance in *Acropora* corals in Ningaloo, and further work is needed to tease apart the relative contribution of both environmental and genetic effects on heat tolerance patterns.

### (d) Host-driven acute heat stress responses in adult corals across reefs

The underlying heat tolerance of the coral holobiont is driven by the coral host, algal symbionts and associated microbiome, and is a complex trait [52]. At both the host and symbiont levels, different individuals within populations can display varying levels of stress tolerance and ability to recover after stress [53]. Interestingly, we found that while the adult hosts exhibited lower overall heat tolerance (figure 4*a*–*d*), their symbionts maintained relatively high photochemical efficiency under heat stress (0.6−0.7) across both species and populations (figure 4*d*). While differing responses are expected between host and symbiont, the more favourable symbiont responses may have been influenced by the relatively low light levels used in our heat stress assay (approximately 60 µmol photons m⁻² s⁻¹; see §5) given that some Symbiodiniaceae taxa are known to maintain high photochemical performance at elevated temperatures under low light [54]. Reef environments are highly dynamic, and light levels in the region have been recorded from as low as 70 µmol photons m⁻² s⁻¹ in March (Sandy Bay near OS [55]) to >480 µmol photons m⁻² s⁻¹ (Tantabiddi, [56]). While we did not take *in situ* light measurements, this lower light level was ultimately chosen to minimize overall stress on the colonies from transport and spawning. In summary, we acknowledge that our experimental conditions likely impacted resulting patterns and that the symbionts may not have experienced as significant stress as the hosts. Importantly, though, the observed patterns have been shown in other species, including adult *Porites astreoides* [57] and *Acropora palmata* [58] colonies. In these studies, adults with the same symbiont communities displayed different heat stress responses, signifying the role of the host in determining phenotypes compared with their symbionts. Drury *et al.* [59] also showed extensive variation in bleaching of *Acropora cerviconis* across distinct colonies with a single dominant symbiont community. Taken together, this shows that the relative contribution of the host coral or symbiont community in driving the heat tolerance response is highly variable, complex and deserves further study.

Finally, we observed relatively low levels of bleaching, but advanced and rapid necrosis followed by mortality in adult corals in both populations (figure 4*b*,*c*). This is likely due to the extreme heat stress (approximately +3°C for OS and +4.5°C for PP above the reported MMM) corals were exposed to. Importantly, the experimental stress coincided with corals’ summer temperature maxima (due to their collection in March), suggesting these corals may have already been exposed to peak annual temperatures. Similar patterns of rapid tissue loss under acute heat stress (+7°C above summer maxima) have been observed in other experimental studies [60,61], and rapid death without bleaching has also been observed in the wild and has been linked to extreme warming conditions [62]. By 2100 on the Ningaloo Reef, mean temperatures are projected to increase from about 1.1°C to >1.2°C, which will exceed present summer maxima for many local reefs [4]. Our results highlight the increasing vulnerability of Ningaloo corals during their critical reproductive window in the warmest summer months.

## 4. Conclusions

Here, we show that after only one generation of breeding along a relatively small geographic distance and temperature differential, selective breeding can enhance offspring heat tolerance in two widespread heat-sensitive species. However, further research is needed to identify the specific drivers of heat tolerance in adult corals and to identify which genetic markers contribute to heat tolerance, and if they are being transferred to offspring. Given future warming scenarios for Ningaloo and projections of ecological functional loss [4], we suggest that selective breeding combined with assisted gene flow could be a feasible conservation tool for enhancing coral heat tolerance along the Ningaloo World Heritage Area.

## 5. Material and methods

### (a) Temperature metrics

To determine thermal regime differences at OS and PP and use as proxy for coral heat tolerance in selective breeding, SST metrics were computed for two timeframes: (i) before recent MHW events (1 January 1985 to 30 September 2010, ‘historical’) and (ii) during and after MHWs (1 October 2010 to 29 December 2022, ‘post-MHW’) [24]. Metrics based on predictors for coral bleaching resistance and heritability of heat tolerance (see [13] included: mean annual SST (SST_mean), s.d. of annual SST (SST_stdev), diurnal SST range (DTR), frequency of thermal anomalies (SSTA_Freq) and cumulative thermal stress in DHWs (TSA_DHW; electronic supplementary material, table S1).

Metrics were derived from the National Oceanic and Atmospheric Administration (NOAA) CoralTemp SST product (v.3.1) [63], NOAA Coral Reef Temperature Anomaly Database (CoRTAD, v.6) [64] and the Integrated Marine Observing System (IMOS) Himawari-8 L3C [65], which offer the highest spatial resolution for WA. Data for the bounded area of interest (i.e. latitudes 23^o^ 33.924′ S to 21^o^ 46.923′ S and longitudes 113^o^ 29.082′ E to 114^o^ 0.078′ E) were downloaded in January 2023 as netCDF files via the THREDDS data server using Python (v.3.9.14 64-bit).

### (b) Site selection and coral collection

The main collection sites were Oyster Stacks (OS, 22^o^ 07.869′ S 113^o^ 52.604′ E) in the northern region and Pelican Point lagoon (PP lagoon, 23^o^ 19.505′ S 113^o^ 46.730′ E) in the southern region (figure 1*a*). For this region, spawning typically occurs within 1−10 days after full moon (7 March 2023) [66]. Collection followed established methods [67]. Full details on the experimental set-up can be found in the electronic supplementary material.

### (c) Selective breeding and larval rearing

At sunset (18.45), within the window of predicted spawning nights, colonies were isolated in individual 60 l polyethylene bins approximately 1 h before the predicted start time for spawning [66]. Full spawning and collection details can be found in the electronic supplementary material.

Reproductive crosses followed such that eggs and sperm were mixed in specific combinations to create distinct families (electronic supplementary material, table S5). Coral fertilizations followed an established method described by Quigley *et al.* [43]. Sperm concentration was estimated based on prior serial dilution trials and optimized for fertilization success for these species [68]. A total of 44 distinct families were produced across the two species, comprising intrapopulation (OS × OS, PP × PP) and interpopulation (OS × PP, PP × OS) crosses, with the maternal colony listed first, followed by the paternal colony. Specifically, *A. tenuis* included 14 of each, and *A. millepora* had 8 of each (electronic supplementary material, table S6). For *A. millepora*, the intrapopulation cross OS × OS was not produced due to insufficient gametes from unique OS colonies.

Eggs and sperm were allowed to fertilize for 3 h, with fertilization success verified by observing initial embryo cleavage at 1.5 h post-fertilization (pf) [69]. When the fertilized eggs from *A. tenuis* crosses reached the four-cell stage, they were moved to separate 15 l transparent cone-shaped polycarbonate tanks (Pentair Aquatic Eco-Systems, USA) and kept at densities of approximately 1–1.2 larvae/ml [15], supplied with 20 μm of filtered seawater (FSW) at 27°C without photosynthetic lights given coral larvae from these species are aposymbiotic. Aeration was turned off until early gastrula stage development (>24 h) [48]. *A. millepora* cultures were reared in 800 ml clamshell-shaped polyethylene containers without flow-through. Complete daily water exchanges using 20 μm FSW at 35 PSU salinity were performed on days 1 and 2 pf as per Marhaver *et al.* [70].

### (d) Larval heat stress experiment

Twenty larvae per family were pipetted into 24 mm netwell inserts with a 74 μm mesh polyester membrane (Corning, USA; electronic supplementary material, table S6). This included *n* = 3 netwell replicates per family per temperature placed into a 6-well floating high-density polyethylene plate as described in Weeryianun *et al.* [35] and Quigley & van Oppen [13]. Control and heat treatments were set to 27.1 ± 0.5°C and 35.5 ± 0.5°C, respectively. The heat treatment temperature was chosen for comparison with previous selective breeding studies on the GBR [11,13,17,20,35].

### (e) Adult heat stress experiment

Multiple colonies for *A. tenuis* (OS = 10, PP = 19) and *A. millepora* (OS = 10, PP = 10) were sectioned into fragments of 5.0 ± 0.7 cm (mean ± s.e.) in length for the adult heat stress experiment after spawning (electronic supplementary material, table S4). Full details on the experimental set-up can be found in the electronic supplementary material. In the heat treatment, 27.1°C water was automatically ramped up in 0.5°C increments per hour until 31.0°C, matching the maximum SST that corresponded to the DHW values during the 2011 MHW in WA [24].

Coral fragments were photographed daily in separate tanks with the same treatment temperatures. Photographs were used to determine survival, tissue necrosis and bleaching scores. Photos were taken using an Olympus Tough TG-6 digital camera (Olympus, Japan) positioned 50 cm from the tanks with fixed settings (ISO 200, focal lens of 25 mm, focal ratio of ƒ2.8 and shutter speed at 1/50 s) and tank illumination (one unit of 72 W aquarium LED lighting Aqua Air 600 set at 50% white). For survival determination, live fragments were scored ‘1’ and dead fragments were scored ‘0’. Dead was defined as bare skeleton with or without microalgae overgrowth. Per cent necrosis was measured using the surface area tool in the Fiji software [71] as described in Quigley *et al.* [48]. Bleaching was determined by assigning scores to fragments relative to the brown hue (D1-D6) from the Coral Health Chart used as proxy for changes in symbiont density and chlorophyll-a content (CoralWatch, Australia; [72]). Bleaching scores of D1 (white) are indicative of a bleached fragment and D6 (brown) of a non-bleached healthy fragment. Scores were assigned to three random points along a vertical axis of each fragment and averaged. Photochemical efficiency of photosystem II in a light-adapted state was measured by the effective quantum yield (∆*F*/*Fm*′) [59] at the start of peak light intensity (10.00) using a DIVING-PAM fluorometer (Walz, Germany) using the following settings: measuring light intensity = 3, saturation pulse intensity = 8, saturation pulse width = 0.8 s, gain = 6 and damping = 2. Measurements were taken consistently 10 mm from the coral tissue and approximately 20 mm above the fragment base. Physiological responses were measured until an average of 50% species mortality was reached during the period from 25 to 31 March (figure 1c).

### (f) Degree heating week calculations for larval crosses

The NOAA Coral Reef Watch DHW formula was used to calculate the accumulated heat stress experienced by corals in our study [63]. Full details on the experimental set-up can be found in the electronic supplementary material. DHWs were calculated based on the projected end-of-the-century regional warming of 1.4°C under the moderate RCP 4.5 using the IPCC Interactive Atlas with the following parameters: CMIP6, SSP24.5, SST variable, recent baseline 1995−2014 and long-term period (2081−2100) [36].

### (g) Statistical analyses

Temperature metrics were calculated in R (v.4.2.2; [73]). For the monthly SST time series, we averaged the monthly SST from daily SST. For SST_mean, we averaged daily SST for each year at each location to obtain the mean annual SST across all years. Similarly, SST_stdev was calculated by averaging the s.d. of annual SSTs. DTR was obtained by subtracting the minimum daily SST from the maximum daily SST and averaging across available years (2016–2022). SSTA_Freq and TSA_DHW were derived directly from the CoRTAD dataset. Except for DTR, all metrics were calculated for the historical and post-MHW periods.

Data normality and homogeneity were checked using Shapiro–Wilk and Levene’s tests, with ‘Shapiro.test’, ‘leveneTest’ and ‘qqnorm’ from the R package ‘stats’ (v.4.2.2) and ‘car’ (v.3.1-2; [74]). Statistical differences were tested using the non-parametric Wilcoxon’s rank-sum test (‘wilcox.test’ from the R package ‘stats’), with a significance level set at 0.05. Temperature metrics were visualized using ‘ggplot2’ (v.3.4.4; [75]).

To assess survival differences in adult fragments between the two populations per species, KM survival curves were used. Survival data were transformed into individual mortality events for KM models. Survival probabilities for control and heat treatments were determined at seven time points. Pairwise comparisons of survival curves were performed using a *post hoc* log-rank test with *p* values corrected using the Bonferroni method [76]. Survival probability with the interaction factor (INT) (temperature × population) was used for comparisons. Survival curves were plotted for each reef and species in the control and heat treatments. R packages ‘survival’ and ‘survminer’ were used for this analysis (v.3.5-7; [77]; v.0.4.9; [78]). The same procedure was applied to evaluate larval survival differences by cross-type and parental origin.

Differences in per cent necrosis, bleaching scores and effective quantum yields at the experimental endpoint were evaluated against temperature treatment and population using GLMMs from the R packages ‘lme4’ (v.1.1-35.1; [79]), ‘glmmTMB’ (v.1.1.8; [80]) and ‘MASS’ (v.7.3-60.0.1; [81]). Temperature and population were converted into an INT, set as a fixed factor in all models. Genotype was the only significant random effect after testing multiple factors. Zero and one inflation were non-significant and dropped from the models. Model assumptions were checked using diagnostic plots and tests from ‘stats’ (v.4.2.2; [73]) and ‘DHARMa’ (v.0.4.6; [82]). Per cent necrosis was converted into proportion in a betaGLMM. A PQL (Gaussian distribution) glmm (PQLglmm) was used for effective quantum yields, and a glmm with negative binomial distribution for bleaching scores. *Post hoc* Tukey’s pairwise comparisons between temperature and population were run on models’ outputs using the function ‘ghlt’ from the ‘multcomp’ package (v.1.4-25; [83]) with *p*-values corrected using the Bonferroni method.

Data for each adult and larval response were summarized with ‘dplyr’ (v.1.1.4 [84]; and plotted using ‘ggplot2’ (v.3.4.4; [75]). Survival of individual larval families was plotted using ‘ggplot2’, and families with significant declines in survival in the heat treatment relative to control were identified using a parametric one-tailed *t*-test using the *t*.test function from ‘stats’ package [73]. *P*-values were corrected for false discovery rate using ‘stats’ (v.4.2.2; [73]). All statistical analyses were carried out using R (v.4.2.2; [73]).

## Data Availability

Data are available on Dryad [85]. Supplementary material is available online [86].
